# How effective is freezing at killing *Anisakis simplex*, *Pseudoterranova krabbei*, and *P. decipiens* larvae? An experimental evaluation of time-temperature conditions

**DOI:** 10.1007/s00436-019-06339-1

**Published:** 2019-05-16

**Authors:** Magdalena Podolska, Bogusław Pawlikowski, Katarzyna Nadolna-Ałtyn, Joanna Pawlak, Katarzyna Komar-Szymczak, Beata Szostakowska

**Affiliations:** 1 National Marine Fisheries Research Institute, Kołłątaja 1, 81-332 Gdynia, Poland; 2 A&A Biotechnology, Aleja Zwycięstwa 96/98, 81-451 Gdynia, Poland; 3 Medical University of Gdańsk, Powstania Styczniowego 9B, 81-519 Gdynia, Poland

**Keywords:** *Anisakis*, *Pseudoterranova*, Freezing, Survival, Herring, Cod

## Abstract

The consumption of raw or inadequately cooked marine fish can lead to several disorders caused by the ingestion of viable anisakid nematodes. Although anisakid larvae can be killed by subzero temperatures, making freezing an important control measure for this potential health hazard, these parasites can survive freezing under some conditions. Therefore, the aim of the present study was to experimentally evaluate the time-temperature conditions needed to kill *Anisakis simplex* and *Pseudoterranova* spp. The effectiveness of freezing was tested on two species of fish: cod, *Gadus morhua* from the North Atlantic, and herring, *Clupea harengus membras* from the southern Baltic Sea. Samples, which comprised skinless fillets of cod (*n* = 40) with visible parasites and whole herring (*n* = 240), were separately frozen at − 15, − 18, or − 20 °C for 24 h, or at − 20 °C for 48 h in the single-compressor freezer and at − 20, − 25, or − 35 °C for 24 h in the double-compressor freezer. After thawing, parasites were stained with malachite green and examined under the microscope for viability. All *A. simplex* and *Pseudoterranova* spp. larvae in cod fillets died at a temperature of − 15 °C or lower. However, freezing did not kill all the *A. simplex* larvae in whole herring: spontaneous movement of these parasites was observed in samples stored in the single-compressor freezer at − 15, − 18, and − 20 °C over 24 h. Our results demonstrate that the freezing procedure must consider both the capability of the freezing device and the nature of the fish product to ensure consumer safety.

## Introduction

The presence of parasites in marine fish is a serious problem for the fishing industry in many countries. Some parasite species may pose a risk to consumers as humans can become accidentally infected with nematodes of the Anisakidae family after consumption of inadequately processed seafood products that contain viable third-stage larvae. *Anisakis simplex* (Rudolphi 1809) and *A. pegreffii* (Campana-Rouget and Biocca 1955) have been reported as causative agents of human infection (Ishikura et al. 1993; Audicana and Kennedy 2008; Mattiucci et al. 2013), but other anisakids (belonging to the *Contracaecum* (Railliet and Henry 1912) and *Pseudoterranova* genera (Krabbe 1878)) are also known to represent a hazard to human health (Shamsi and Suthar 2016; Mehrdana et al. 2014; Mattiucci et al. 2013; Shamsi and Butcher 2011; Torres et al. 2007). Symptoms include nausea, diarrhea, vomiting, and intense abdominal pain (Ishikura et al. 1993; Hochberg and Hamer 2010). Human health may also be compromised by allergic reactions to parasite antigens (hypersensitivity) (Audicana and Kennedy 2008; Valero et al. 2003; Mehrdana and Buchmann 2017).

Zoonotic nematodes of the Anisakidae family are widespread in a variety of fish species worldwide (Mattiucci and Nascetti 2008; Buchmann and Mehrdana 2016). The European Food Safety Authority (EFSA) states that all wild caught seawater and freshwater fish must be considered at risk of containing viable parasites of human health concern if these products are to be eaten raw or almost raw (EFSA 2010). Treatment to kill viable parasites in fishery products intended for human consumption is mandatory in many EU countries, USA and Canada (EFSA 2010). EU Regulation No. 1276/2011 (European Commission 2011) requires that food business operators must ensure that the raw material or finished product undergoes a freezing treatment in order to kill viable parasites that may be a risk to the health of the consumer. For parasites other than trematodes, the freezing treatment must consist of lowering the temperature in all parts of the product to at least − 20 °C for not less than 24 h, or to − 35 °C for not less than 15 h. The U.S. Food and Drug Administration (FDA) recommends that all shellfish and fish intended for raw consumption should be blast frozen to − 35 °C or below for 15 h or be regularly frozen to − 20 °C or below for 7 days (FDA 2011).

Thermal processing of fishery products is the most effective way of killing anisakid larvae. On the other hand, some studies have revealed that anisakid nematodes possess a high tolerance to a wide temperature range and have the ability to survive freezing at temperatures well below zero (Adams et al. 2005; Oh et al. 2014; Wharton and Aalders 2002). This phenomenon is attributed to the presence of trehalose, which can act as a cryoprotectant (Wharton and Aalders 2002).

The effectiveness of the freezing of fish products depends on many factors, e.g., species of fish (fatty or lean), type of raw material (fillets or whole fish), mass and volume of fish product, power of the freezer unit, and how full it is (Deardorff and Throm 1988; Wharton and Aalders 2002; Adams et al. 2005). Therefore, the holding time required to reach the target temperature inside the product varies depending on the device used, although it generally takes many hours. The question arises: how long should the product be held at the set freezing temperature (from the moment of placement in, until its removal from, the freezer) to meet the EU criteria? The legislative text specifies the temperature and time needed for the treatment to kill viable parasites, but many small-scale fish processors use freezing temperatures other than those (i.e., − 20 and − 35 °C) recommended by the EU regulations. In addition, fish and fish products are often frozen by householders in domestic freezers, which operate at a low cooling capacity and therefore cannot attain such low temperatures. According to Sanchez-Alonso et al. (2018), the use of household freezers represents the highest risk of inappropriate application of EU recommendations.

Reports on the impact of freezing on the survival of anisakid nematodes are sometimes contradictory. The early studies of Gustafson (1953) showed that freezing for 24 h at − 17 °C destroyed nematodes, and Lanfranchi and Sardella (2010) revealed that 100% of *Anisakis* larvae from Argentinean fish died after 24 h at − 20 °C. However, different results were obtained by Oh et al. (2014), who investigated the effect of freezing on squid and pollock tripe into which *A. simplex* larvae had been inoculated: viable larvae (1.7 to 3.9%) were found after 1 day of storage at − 20 °C. Similarly, Adams et al. (2005) reported that *A. simplex* larvae in arrowtooth flounder (*Atheresthes stomia*) can survive freezing at − 20 °C for up to 48 h.

The aim of the present study was to experimentally evaluate the time-temperature conditions necessary to kill anisakid larvae (*A. simplex* and *Pseudoterranova* spp.) in two types of freezer with different rates of temperature change. The effectiveness of the freezing process was tested on two species of fish known to be naturally infected with anisakids: cod, *Gadus morhua* (L.) from the North Atlantic (Gay et al. 2018), and herring, *Clupea harengus membras* (L.) from the southern Baltic Sea (Horbowy and Podolska 2001). These two fish species are commercially exploited and processed and are very popular among consumers. The viability of anisakid larvae after freezing was assessed based on their mobility (EFSA 2010) and susceptibility to staining with malachite green. Application of this dye allows dead and viable nematodes to be distinguished (Leinemann and Karl 1988).

## Materials and methods

Two species of fish were selected for the study: (1) *G. morhua*, obtained from catches in the North Atlantic (Divisions 27.2.a.1 and 27.2.a.2 of FAO Major Fishing Area 27), which was selected due to the high level of natural infection of this species with Anisakidae, particularly cod worm (seal worm) *Pseudoterranova* spp. (Mehrdana et al. 2014, Buchmann and Mehrdana 2016; Gay et al. 2018). Although a high level of *G. morhua* infection with *Contracaecum osculatum* has also been reported in recent years, this species of parasite was not included in our study because it is only occasionally present in muscle tissue (*C. osculatum* larvae occur mainly in the liver of fish). (2) *C. harengus membras* caught in the southern Baltic Sea (Division 27.3.d of FAO Major Fishing Area 27, ICES Subdivision 24), which is often naturally infected with herring worm (whale worm) *A. simplex* (Horbowy and Podolska 2001; Skov et al. 2009).

Two freezers with different rates of temperature change were used: a single-compressor freezer (model LGT-4725, Liebherr, Germany), with a conventional (static) cooling system (compressor power 433 W), without air circulation inside the refrigeration compartment; and a double-compressor freezer (model MDF-U443-PE, Panasonic, Japan) with a cascade cooling system (compressor power 400 and 750 W) and fan-forced air circulation in the refrigerator for precise temperature uniformity.

Samples comprised pieces of skinless *G. morhua* fillets (thickness 1.5–2 cm; *n* = 40) with visible parasites and whole (ungutted) *C. harengus membras* (length 26–31 cm; *n* = 240). Fresh *G. morhua* fillet samples were obtained directly from the production line of the fish processing plant. *C. harengus membras* was purchased from fishermen, immediately after capture, and fish were transported on ice to the laboratory. Fish samples were kept in a refrigerator at 4 °C (for 12 h) prior to the freezing experiment. *G. morhua* fillets were placed separately in polyethylene bags and exposed to temperatures of − 15 and − 20 °C in the single-compressor freezer and − 18 and − 25 °C in the double-compressor freezer for 24 h. Samples of whole *C. harengus membras* were placed in polyethylene bags (two individuals per bag) and held at the following set temperatures: − 15, − 18, or − 20 °C for 24 h and − 20 °C for 48 h in the single-compressor freezer; or at − 20, − 25, or − 35 °C for 24 h in the double-compressor freezer. During the entire freezing process, both the internal temperature of samples (measured at the center of the thickest part of the fillet or fish) and the ambient freezer temperature were recorded with wireless Track Sense Pro data loggers (Ellab, Denmark), which are double-rigid temperature sensors with an accuracy of 0.05 °C at 15 min intervals. Ellab ValSuite ver. 4.0 software was used to record and analyze the parameters of the freezing process. The freezing rate was defined according to the following equation, based on the time taken to cross the temperature zone of maximum ice crystal formation (Kono et al. 2017):

$$ \mathrm{Freezing}\ \mathrm{rate}\ \left({}^{{}^{\circ}}\mathrm{C}/\min \right)=\left({T}_2-{T}_1\right)/\left({t}_2-{t}_1\right) $$

where*T*_1_ = 0.0 °C *T*_2_ = − 5.0 °C*t*_2_ − *t*_1_ is the time taken for the core sample temperature to change from 0.0 to − 5.0 °C.

After thawing, forceps were used to carefully remove nematodes from *G. morhua* fillets and *C. harengus membras* body cavities. Nematodes were identified to the genus level based on anatomo-morphological descriptions given by Berland (1961, 1989) and Fagerholm (1982). Each parasite isolated was analyzed for viability according to EFSA (2010). Thus, larvae that moved spontaneously or after stimulation with tweezers were considered alive. Motionless larvae were incubated in a thermoblock at 37 °C for 1.5 h, after which their mobility was evaluated again. Larvae that were still motionless after incubation were stained with malachite green. This dye stains dead larvae intensely, whereas viable individuals remain colorless (Leinemann and Karl 1988). The dye was prepared by dissolving 0.1-g malachite green in 10-mL distilled water. The staining mixture consisted of 10-mL malachite green solution and 90-mL 0.5% pepsin solution (dissolved in 0.85% NaCl) at pH 2 (adjusted using HCl). Parasites were placed in Eppendorf tubes containing 1.5-mL staining mixture and were incubated in a thermoblock at 37 °C for 1.5 h. This time was sufficient for intense staining of dead or severely damaged parasites and allowed us to assess the survival of larvae shortly after the freezing experiment. Finally, the larvae were rinsed with 0.9% NaCl and observed under a microscope (AxioZoom V16, Carl Zeiss, Germany) to assess the degree of damage after freezing.

Randomly selected subsamples of the above parasites (100 *Anisakis* larvae from *C. harengus membras* body cavities, 10 *Anisakis*, and 94 *Pseudoterranova* larvae from *G. morhua* fillets) were subjected to molecular identification. DNA was isolated using a Genomic Mini Kit (A&A Biotechnology, Gdynia, Poland) according to the manufacturer’s instructions. The target of analysis was the internal transcribed spacer 1 (ITS-1) of the ribosomal DNA (rDNA). The amplification was performed using NC5 (forward) 5′ GTA GGT GAA CCT GCG GAA GGA TCA TT 3′ and NC13 (reverse) 5′ GCT GCG TTC TTC ATC GAT 3′ primers (Zhu et al. 2000). The reaction mixture consisted of 25-μL PCR Mix HGC Plus (ready-to-use PCR mixture containing Taq DNA polymerase, PCR buffer, MgCl_2_, and dNTPs; A&A Biotechnology), 2-μL each primer (in concentration 10 μM), and 5-μL DNA template, supplemented with deionized water up to 50 μL. The PCR reaction conditions were as follows: 3 min at 94 °C (initial denaturation) followed by 30 cycles of 30 s at 94 °C, 30 s at 55 °C, 30 s at 72 °C, and a final extension step of 5 min at 72 °C. The PCR products were sequenced directly using standard procedures. The sequences obtained were analyzed using GeneStudio Pro Software (GeneStudio, Inc., Suwanee, GA, USA).

## Results

**Fig. 1:**
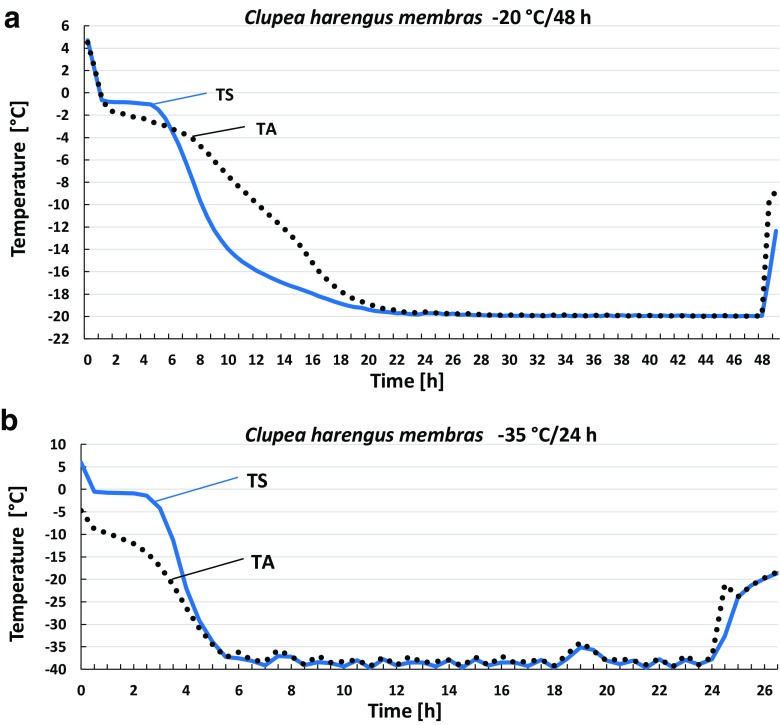
Freezing curves of *C. harengus membras* samples. **a** Single-compressor freezer (− 20 °C/48 h). **b** Double-compressor freezer (− 35 °C/24 h). TS: internal temperature of samples (solid line); TA: freezer ambient temperature (dotted line)

The freezing process varied depending on the type of fish sample, the freezing device used, and its target temperature. Typically, freezing rates of *G. morhua* fillets were faster than *C. harengus membras* samples (whole fish), and there were marked differences between the results with two freezers. In the single-compressor freezer, fillets reached the target temperatures of − 15 and − 20 °C after 6 h 15 min and 9 h 30 min, respectively. In contrast, a temperature of − 18 °C was achieved in fillets after 3 h 45 min in the double-compressor freezer, while the time required to reach − 25 °C was 2 h 45 min. This latter, shorter time occurs because both compressors are activated when lower target temperatures are set. *C. harengus membras* samples placed in the single-compressor freezer were frozen to − 15 °C after 15 h 15 min, while freezing to − 18 °C took 20 h 15 min. The time required to reach the target temperature of − 20 °C in samples was more than twice as long in the single-compressor (over 23 h) as in the double-compressor device (10 h 15 min). Thus, the time required to reach − 20 °C in *C. harengus membras* samples held in the single-compressor freezer was almost 24 h, giving a freezing rate of 0.01 °C/min. With a holding time in the single-compressor freezer of 24 h, the time *C. harengus membras* samples spent at the target temperature was only 15 min. In the double-compressor freezer, a temperature of − 25 °C was achieved in samples after 6 h 15 min, while freezing to − 35 °C took only slightly longer (6 h 45 min). A faster freezing rate of 0.14 °C/min was recorded during freezing of *G. morhua* fillets at − 35 °C in the double-compressor freezer, where the target temperature was achieved in samples after 2 h 45 min. The freezing parameters for fillets of *G. morhua* and *C. harengus membras* are given in Table 1a,b. Selected freezing curves for *C. harengus membras* samples held in the single-compressor (− 20 °C/48 h) and double-compressor freezers (− 35 °C/24 h) are presented in Fig. 1.

**Table 1:** Freezing parameters for *Gadus morhua* from the North Atlantic (a) and *Clupea harengus membras* (b)

(a) *G. morhua*—fillets							
Freezer	Liebherr (single compressor)		Panasonic (double compressor)				
Target temperature [°C]	− 15	− 20	− 18	− 25			
Duration of freezing [h]	24	24	24	24			
Time to reach target temperature^a^ [h, min]	6 h 15′	9 h 30′	3 h 45′	2 h 45′			
Effective freezing time at target temperature^a^ [h, min]	17 h 45′	14 h 30′	20 h 15′	21 h 15′			
Freezing rate [°C/min]^b^	0.02	0.02	0.06	0.14			
(b) *C. harengus membras*							
Freezer	Liebherr (single compressor)				Panasonic (double compressor)		
Target temperature [°C]	− 15	− 18	− 20	− 20	− 20	− 25	− 35
Duration of freezing [h]	24	24	24	48	24	24	24
Time to reach target temperature^a^ [h, min]	15 h 15′	20 h 15′	23 h 45′	23 h 30′	10 h 15′	6 h 15′	6 h 45′
Effective freezing time at target temperature^a^ [h, min]	8 h 45′	3 h 45′	15′	24 h 30′	13 h 45′	17 h 45′	17 h 15′
Freezing rate [°C/min]^b^	0.01	0.01	0.01	0.01	0.02	0.04	0.07

^a^Core temperature of the sample

^b^Freezing rate [°C/min] = (*T*_2_ − *T*_1_)°/°(*t*_2_ − *t*_1_), where *T*_1_ = 0.0 °C and *T*_2_ = − 5.0 °C; *t*_2_ − *t*_1_ is the time taken for the core sample temperature to change from 0.0 to − 5.0 °C

In total, the *G. morhua* fillets contained 990 larvae of the genus *Pseudoterranova*, while *Anisakis* larvae, which were also found in fillets, were less numerous (*n* = 72) (Table 2). A total number of 1267 *Anisakis* larvae were found in *C. harengus membras* body cavities. *Hysterothylacium* larvae, which can be identified morphologically to the genus level, were not found in *C. harengus membras*. The only species molecularly identified from the body cavity of *C. harengus membras* was *A. simplex* sensu stricto (s.s.). Molecular identification revealed the presence of three species of anisakids in fillets of *G. morhua*. The major sibling species identified within the *P. decipiens* complex was *P. krabbei* (75 individuals) followed by *P. decipiens* s.s. (19 individuals). Ten individuals were assigned to the species *A. simplex* s.s.

**Table 2:** Time-temperature conditions for freezing and the number of anisakid larvae in fillets of *Gadus morhua* from the North Atlantic

Freezer	Duration (h)	Temperature (°C)	Fillets of cod		Number of larvae			
			Number	Average mass (g)	*A. simplex* s.s.		*Pseudoterranova* spp.	
					Total^a^	Active (live)	Total^a^	Active (live)
Liebherr	24	− 15	13	107.8	7	0	218	0
(single compressor)		− 20	7	292	33	0	243	0
Panasonic	24	− 18	13	153.1	14	0	451	0
(double compressor)		− 25	7	260.9	18	0	78	0
Sum			40	181.5	72	0	990	0

^a^All individuals found in samples

Only larvae present in the body cavities of *C. harengus membras* were analyzed in this study. Detection of larvae present in the flesh requires the digestion of muscle tissue in artificial gastric juice for a minimum of 24 h. To obtain reliable results, the viability of the larvae should be assessed immediately after the freezing experiment, without additional treatment. Another commonly used technique for detecting larvae in the muscle tissue of fish is candling, but in the case of *C. harengus membras*, this method is ineffective due to its dark, opaque flesh.

All individuals of *Pseudoterranova* spp. and *A. simplex* s.s. from *G. morhua* fillets were motionless after freezing (≤− 15 °C for 24 h) and thawing, even after stimulation with tweezers; furthermore, no mobility was observed after incubation at 37 °C. Application of dye (malachite green) showed staining of each parasite examined, confirming that all *Pseudoterranova* spp. and *A. simplex* s.s. larvae were dead.

In contrast, after freezing some  *A. simplex* s.s. larvae found in *C. harengus membras* body cavities were still alive. Spontaneous movement was observed in 12 parasites (0.9%) held in the single-compressor freezer for 24 h. Eight larvae (2.9%) kept at − 15 °C for 24 h were active immediately after thawing. A few larvae survived at − 18 °C (two individuals, 0.8%, kept for 24 h) and − 20 °C (two individuals, 4.7%, kept for 24 h) and showed spontaneous mobility after thawing. All parasites stored in the single-compressor freezer at − 20 °C for 48 h and in the double-compressor device at ≤− 20 °C for 24 h were motionless after thawing. Stimulation with tweezers and incubation at 37 °C did not provoke any activity (Table 3).

**Table 3:** Time-temperature conditions for freezing and the number of *A. simplex* s.s. larvae in *Clupea harengus membras*

Freezer	Duration (h)	Temperature (°C)	Number of herring		Number of *A. simplex* s.s. larvae	
			Examined	Infected	Total^a^	Active (live)
Liebherr (single compressor)	24	− 15	60	42	272	8
		− 18	60	50	239	2
		− 20	40	19	43	2
	48	− 20	60	34	103	0
Panasonic (double compressor)	24	− 20	60	45	245	0
		− 25	60	44	199	0
		− 35	60	35	166	0
Sum			400	269	1267	12

^a^All individuals found in samples

**Fig. 2:**
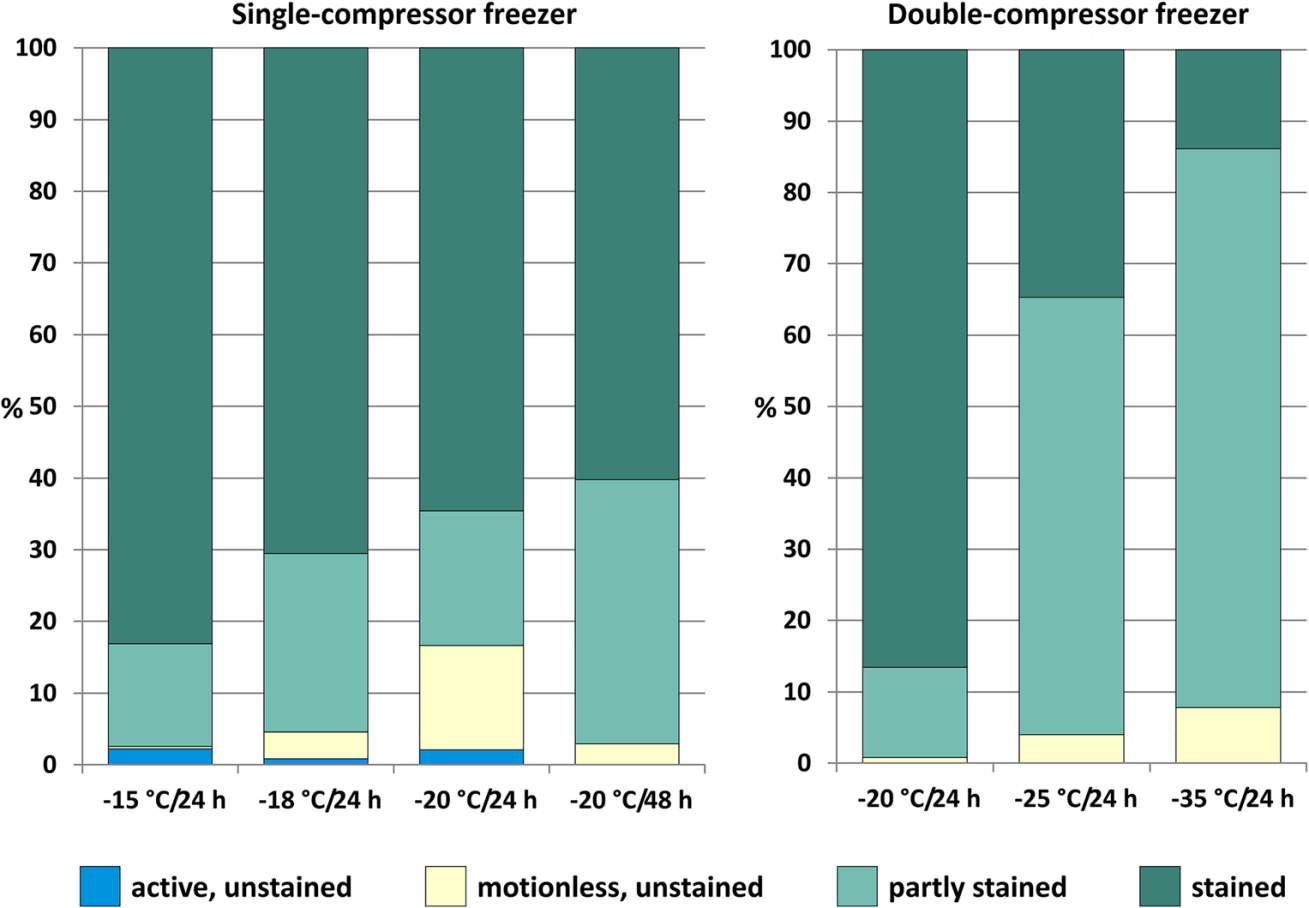
Mobility and coloration of *A. simplex* s.s. larvae obtained from *C. harengus membras* samples after freezing

Application of malachite green after thawing of *C. harengus membras* samples allowed rapid identification of the apparently dead parasites. The majority of *A. simplex* s.s. larvae frozen in the single-compressor freezer were intensely stained. Some parasites were only partly stained (14–37%), while a few individuals (in each sample) remained colorless (up to 16% at − 20 °C/24 h). All unstained larvae were motionless (Fig. 2). In contrast, the percentage of intensely stained larvae frozen in the double-compressor freezer decreased with decreasing freezing temperature. Only 14% of *A. simplex* s.s. larvae were clearly stained after freezing at − 35 °C (24 h). High percentages of partly stained (78%) larvae were observed under these time-temperature conditions. In the majority of larvae held in the double-compressor freezer, the only stained part of the body was the ventriculus.

**Fig. 3:**
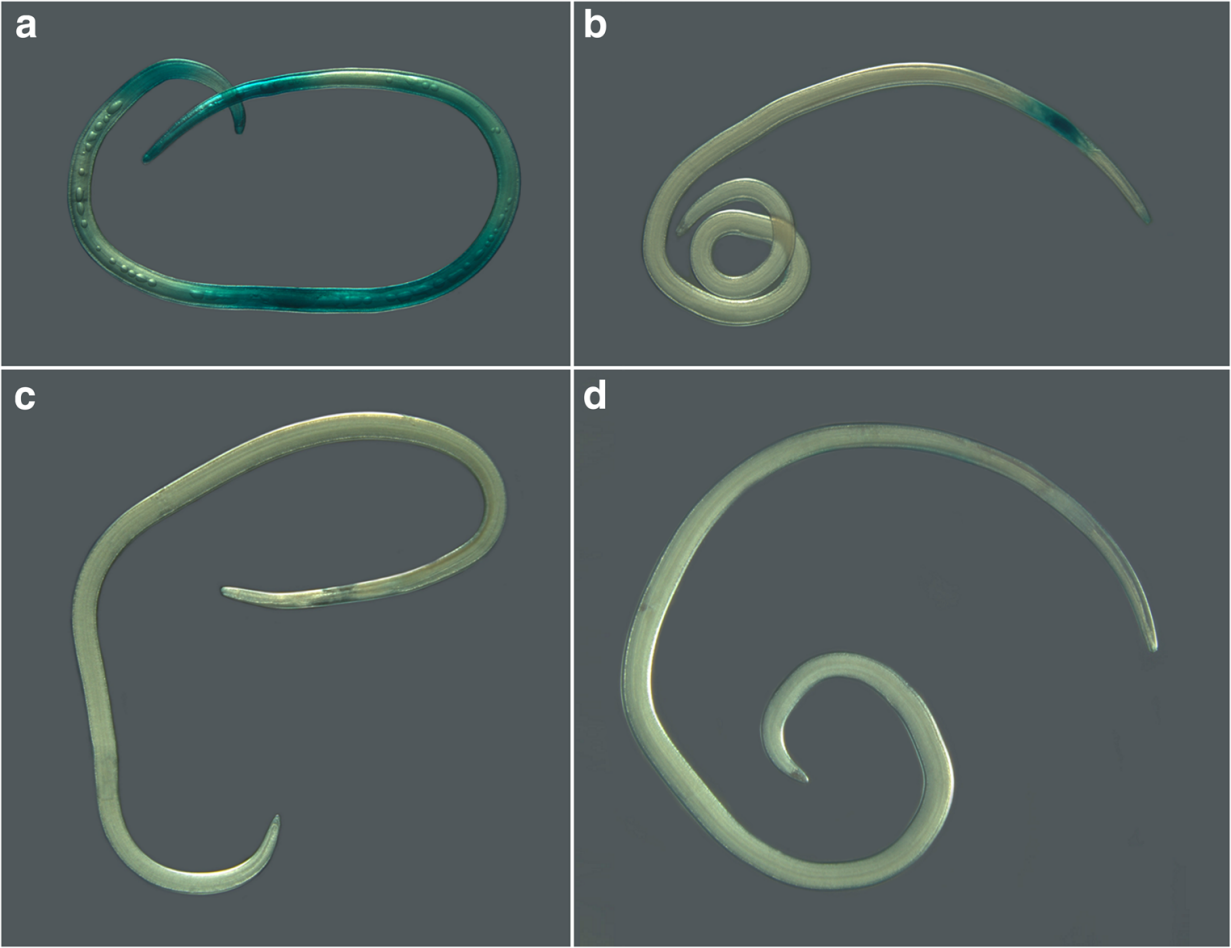
*A. simplex* s.s. larvae after freezing, thawing and staining with malachite green. **a** Blisters (− 20 °C/48 h). **b** Bloated ventriculus (− 35 °C/24 h). **c** Damaged ventriculus, larva remained colorless (− 20 °C/48 h). **d** Damaged tail, larva remained colorless (− 35 °C/24 h)

Microscopic observation of *A. simplex* s.s. larvae after staining revealed a number of different types of body damage. Intensely stained individuals usually showed severe damage to the body structure, e.g., cuticle breakage and blisters. Lesions of the ventriculus (e.g., bloating) were the most commonly observed damage in partly stained nematodes. Some of the larvae that were frozen in the double-compressor freezer and remained colorless after staining did not show any apparent damage. Examples of *A. simplex* s.s. tissue damage after freezing are shown in Fig. 3.

## Discussion

In the present study, *A. simplex* s.s. was the only species molecularly identified from the body cavity of *C. harengus membras.* Nematodes isolated from *G. morhua* fillets represented *P. decipiens* s.s., *P. krabbei* and *A. simplex* s.s. This is in line with previously published genetic data according to which *A. simplex* s.s. is the only *Anisakis* species recorded in the Baltic (Mattiucci et al. 1997). According to Gay et al. (2018), the major species isolated from the fillets and the viscera of *G. morhua* from the Northern North Sea was *A. simplex* s.s. (99.8%), while only three individuals of *A. pegreffii* were identified in fillets. Two sibling species of *P. decipiens* complex (*P. decipiens* s.s. and *P. krabbei*) were identified in fillets of *G. morhua* from the Northern North Sea (Gay et al. 2018).

The freezing rate is considered the most important factor influencing the size and location of ice crystals during freezing (Dalvi-Isfahan et al. 2017). Fast freezing produces small ice crystals, resulting in less tissue damage than a slow freezing rate, which usually results in the formation of more damaging, larger ice crystals (Erikson et al. 2016). The process of ice crystal formation is important not only for the quality of the fish product but also for the survival of anisakid nematodes during freezing (Wharton and Aalders 2002).

The results of our experiment did not demonstrate a difference in freezing tolerance of *A. simplex* s.s. and *Pseudoterranova* spp. All anisakid larvae in *G. morhua* fillets died at a temperature of − 15 °C or lower. However, this lack of difference in freezing tolerance requires further confirmation through future studies specifically designed for that purpose. According to Stormo et al. (2009), *P. decipiens* larvae may have a freeze tolerance similar to that of *A. simplex*. In contrast, the study of Lanfranchi and Sardella (2010) revealed that 100% of *Anisakis* sp. larvae survived 5 h at temperatures in the range of − 18 to − 22 °C, whereas larvae of *Pseudoterranova* sp. died within the first 3 h, which may suggest a lower resistance to freezing of the latter species.

Interpretation and comparison of results obtained by other authors are often difficult due to the unavailability of detailed data on the conditions of the freezing process used. Even if the same temperature and holding time are used in separate experiments, the results obtained can vary depending on the type of freezer, the freezing rate, and the nature of the frozen sample. In our approach, we assessed the impact of the following parameters on the survival of anisakid nematodes: temperature set in the freezer, time for the internal temperature of the sample to reach the set temperature, type of freezer (single- vs double-compressor), and type of raw material (skinless fillets vs whole fish; fatty vs lean fish). We demonstrated that *A. simplex* s.s. larvae survived in *C. harengus membras* held in a single-compressor freezer at − 20 °C for 24 h. In this freezing device, the time to reach target temperature in the sample was over 23 h. Under the same time-temperature conditions, but in a double-compressor freezing device (with a freezing rate twice as high), no viable parasites were recovered from *C. harengus membras*. In this case, the time needed to achieve the target temperature was much shorter (10 h 15 min). The freezing rate depends also on how full the freezer is, the mass of the fish, and the sample size. Wharton and Aalders (2002) found that the core of 20-kg containers of fish did not achieve ambient temperatures of − 35 °C after 28-h exposure, whereas *C. harengus membras* samples frozen during the present study reached − 35 °C after 6 h 45 min, which resulted in an effective freezing time lasting more than 15 h. No *A. simplex* larvae were viable after freezing under these time-temperature conditions. This result is in accordance with the finding of Deardorff and Throm (1988) that blast freezing to at least − 35 °C for 15 h effectively killed larval *A. simplex.* Other factors can affect the survival of anisakid nematodes, such as species of fish or type of raw material. Whole fish, still containing their viscera, might offer better physical protection to nematodes during freezing than gutted and headed fish (Deardorff and Throm 1988; Adams et al. 2005). Our results revealed that in the same (single-compressor) freezer, the effective freezing time for skinless *G. morhua* fillets at − 15 °C was almost twice as long (17 h 45 min) as for whole (ungutted) *C. harengus membras* (8 h 45 min).

EU Regulation No. 1276/2011 recommends freezing at − 20 °C or below for 24 h, or − 35 °C or below for 15 h, to kill parasites. During our investigation, several time-temperature conditions were tested, including − 25 °C, which is commonly used in fish processing plants, as well as − 15 and − 18 °C, which are typical of domestic freezers. Some *A. simplex* s.s. larvae subjected to freezing in the single-compressor device survived at − 15 and − 18 °C, which has implications for domestic freezer use. According to Sanchez-Alonso et al. (2018), the ability of some *Anisakis* larvae to survive freezing at these temperatures poses a risk to households, because a significant percentage of domestic freezers cannot attain the minimum temperature of − 20 °C recommended by the EU.

Live and dead anisakid larvae can be distinguished by observation of parasite motility (EFSA 2010), by the fluorescence of dead larvae excited by UV radiation (Rodriguez-Mahillo et al. 2008; Vidacek et al. 2010), and by staining with different dyes (Leinemann and Karl 1988). However, the utility of some of these procedures is questionable. In our study, spontaneous movement was observed in some *A. simplex* s.s. larvae immediately after thawing, and therefore clearly, these individuals were viable. Staining with malachite green is also useful for preliminary screening of apparently dead larvae. However, some experimentally frozen *A. simplex* s.s. larvae remained colorless after staining with malachite green despite being motionless. The majority of these were intact and without apparent damage to the body structure, as viewed under the light microscope. The highest percentage (16%) of unstained larvae was reported in *C. harengus membras* samples frozen at − 20 °C for 24 h in the single-compressor freezer. In this case, the limited time of exposure to the target temperature (only 15 min) might be insufficient to cause the structural damage necessary for the malachite green stain to penetrate the parasite tissues.

A high proportion of unstained larvae was also observed in *C. harengus membras* samples frozen in the double-compressor freezer, and the uptake of dye by *A. simplex* s.s. decreased with decreasing freezing temperature. The highest percentage of partly stained individuals (78%) occurred in larvae frozen at − 35 °C. Consequently, rapidly frozen parasites are likely to suffer less cellular damage and would be expected to absorb malachite green to a lesser extent than larvae frozen slowly. Lesion of the ventriculus (bloating) was the most commonly reported damage in partly stained nematodes. Moreover, the ventriculus was the only part of the body that was stained in the majority of larvae held in the double-compressor freezer. These findings suggest that this part of the *A. simplex* s.s. body is the most sensitive to freezing.

Among the experimentally tested time-temperature conditions (− 15, − 18, or − 20 °C for 24 h, − 20 °C for 48 h in the single-compressor freezer, and − 20, − 25, or − 35 °C for 24 h in the double-compressor freezer), only two met the criteria listed in the EU regulations: freezing of *C. harengus membras* samples in the single-compressor freezer at − 20 °C for 48 h, where the effective freezing time at the target temperature lasted > 24 h, and freezing in the double-compressor device at − 35 °C for 24 h, with an effective freezing time of > 15 h. It is important to note that the holding time of the product at the required freezing temperature should be sufficiently long to kill all viable anisakid nematodes.

The freezing process in the laboratory, which of necessity takes place on a small scale (e.g., single fillets), clearly differs from freezing on an industrial scale, where fish are often frozen in blocks. Nevertheless, it should be possible to monitor the freezing process and its effectiveness in almost every processing plant. By recording the parameters of the freezing of fish products (e.g., using loggers or thermocouples), it is possible to assess under which conditions the required temperature will be reached in all parts of the product and maintained for a sufficient length of time. As a result, the freezing process can be optimized to ensure that products are safe for consumers.
